# Offensive Breath

**Published:** 1871-03

**Authors:** J. A. Munk

**Affiliations:** Lindsey, O.


### OFFENSIVE BREATH.

BY J. A. MUNK, M. D., LINDSEY, O.

The popular term, "bad breath," is a very significant expression for this unpleasant condition. What is more offensive to the acute olfactory sense than a fetid breath? It engenders a feeling of aversion and disgust, which is not readily overcome. It serves as a means in many instances of estranging intimate friends.

A consideration of this subject may not properly belong to medicine; yet it deserves the physician's attention, for he is frequently applied to in these cases for relief.

Naturally, the breath of a young healthy person possesses an agreeable flavor and sweetness; but which with age becomes lost. In the majority of cases, pernicious habits or disease occasion a tainted breath. The most commonly indulged evil practices are the use of ardent spirits and the odious weed, tobacco. It not only obtrudes uncomfortably upon associates, but they are imminently prejudicial to the physical well-being. Through their narcotic and poisonous properties they exert a very deleterious influence upon the nervous system. The nice sensibility of the nerves becomes blunted in degree, even to a disregard for cleanliness and refinement. It is utterly impossible for an inveterate chewer or smoker to retain a beautiful set of dentals, much less a pure breath. Great care should be exercised in keeping the mouth free from all extraneous substances. After each meal, a quill or ivory toothpick should be used to remove any aliment that may have become lodged in the teeth during the process of mastication, and the mouth rinsed with tepid soft water. Every night, previous to retiring, the teeth should be cleansed with a soft tooth brush and water. As a rule, tooth pastes and powders should be eschewed as harmful agents. If a dentifrice is desired, a little fine toilet soap, or charcoal reduced to an impalpable powder, may be used. This is all that will be required. Decayed teeth are a very prolific source of mephitic breath. As soon as it is ascertained that a tooth is affected, it should have immediate attention, and receive the services of some competent dentist to extract, fill, or treat in any manner its condition demands.

Carious teeth are often the source of serious functional and general disturbance. It sometimes occurs that persons with a number of defective teeth are constantly ailing with either gastric or nervous troubles, when, upon a removal of these unsound members, all the unpleasant symptoms promptly disappear.

It may be well to give a word of caution in regard to diet; by irregularities in eating, the digestive functions become impaired, and for want of proper digestion, the aliment undergoes zymotic change, during which process noxious gases are evolved, and cause a foul breath. When cases arise from disease, it is either of the stomach, lungs, or the respiratory passages. In these cases a physician should be consulted at once; for with proper medical aid, patience and perseverance, the patient can hope to obtain relief. Many substances are in vogue to sweeten the breath, and to disguise any unpleasant scent, as of spirits, tobacco, etc. With the vulgar it is customary to use some pungent aromatic, as cloves, etc., but this savors too strongly of the drinking bar to be used by any but tipplers. The following used as an occasional mouth wash, will be found excellent: Take Chlorate of Potash, three drachms, dissolve in eight ounces of rose or other medicated water. As an article with which to flavor the breath, there is probably nothing equal to the Wild Ginger, (*Asarum Canadensis*). It is used by chewing a small portion of the root, or if in powder it can be made into a lozenge. It imparts to the breath an agreeable spicy aroma, and is in every respect *a la bonne heure.*

